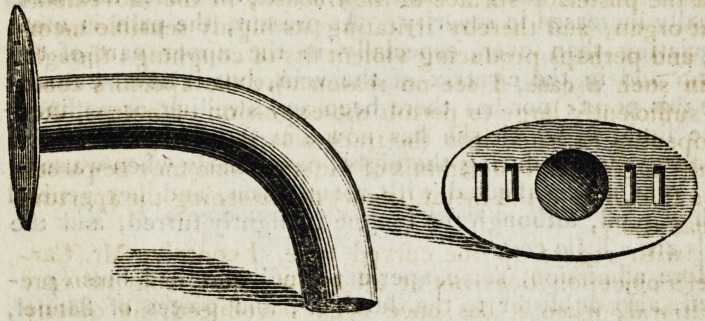# Case of Diseased Larynx, in Which Tracheotomy Was Performed

**Published:** 1825-07

**Authors:** William James Goodeve

**Affiliations:** Member of the Royal College of Surgeons; Surgeon to the Clifton Dispensary, and Lecturer on Anatomy and Surgery.


ART. V.—*Case of Diseased Larynx, in which Tracheotomy was performed.* By WILLIAM JAMES GOODEVE, Esq. Member of the Royal College of Surgeons; Surgeon to the Clifton Dispensary, and Lecturer on Anatomy and Surgery.

J. L. æt. thirty-six, living in a damp unhealthy situation, very much exposed to night air, and liable to frequent rheumatic attacks, suffered for several months, in 1820, with ulcerated sore throat, which was suspected to be syphilitic, and which, at last, healed under the use of nitric acid and small doses of mercury. Ulceration has occasionally returned, and has been removed by the same remedies.

About a twelvemonth ago, a swelling took place at the sternal end of the cartilage of the fourth right rib. This swelling subsided under the use of mercury, in such doses as slightly to affect the mouth. Not long afterwards, a similar painful swelling, but unattended with much inflammation of the integuments, occurred on the upper bone of the sternum: this also subsided, under the exhibition of ten grains of pil. hydrarg. every night for three weeks.

Within the last three months, the membrane of the larynx has been very frequently affected with inflammation, giving rise to hoarseness of the voice and considerable difficulty in respiration. At one period, there appeared a very active inflammation about the cricoid cartilage, which was much swelled, and very tender to the touch. Leeching and blistering seemed to subdue this inflammation; and, under the use of pil. hyd. and decoct. sars. c., great amendment took place. However, some return of the hoarseness and difficulty in respiration followed, and two or three most alarming paroxysms of suffocation occurred. In one of these, when he appeared just on the point of being suffocated, and after having endured for some hours extreme suffering, a piece of bone was expelled through the glottis, with amazing violence. This was at first supposed to have been a portion of ossified cricoid cartilage; but more accurate examination of its form and cancellous structure, convinced me that it came from the upper and back part of the triangular bone of the sternum, just between the clavicular articulations.

After parting with this substance, his voice and respiration gradually improved. To this amendment I suspected that the frequent inhalation of the fumes of cinnabar, which he was recommended to try at this time, in some degree contributed; and at the same time his general health became very much better, under the use of five grains pil. hyd. o. n., and a pint of decoct. sars. c. ter die. But, while yet using these remedies, there occurred, somewhat suddenly, about a month ago, a return of inflammation in the larynx, giving rise to considerable hoarseness, and at intervals very severe dyspnœa. Cathartic and sudorific medicines were prescribed; a blister was applied over the larynx and trachea, and kept open by means of savin cerate, and he was directed to inhale steam seven or eight times in the course of the day. The inhalation afforded him, *pro tempore*, some relief; but every two or three days he experienced, for an hour or two together, very considerable difficulty in breathing. His voice became gradually more affected, and these attacks of dyspnœa more frequent. There was a continual sense of tightness and constriction about the upper end of the sternum, but hardly any cough, and no pain in the chest. To relieve this oppression, a large blister was placed over the sternum, and kept open about a month. In spite of these measures, the disease in the larynx gained ground, and the threatenings of suffocation became more frequent and alarming.

Noting the advance of these bad symptoms, I looked to tracheotomy, as affording him the only chance of escape from the very imminent hazard to which he was so often exposed; and yet I felt some distrust about the operation, from not knowing exactly to what distance the disease of the membrane might have extended; and I was inclined to fear, from the uneasiness he felt at the bottom of the trachea, that all was not right there: indeed, I rather thought that more bone might be finding its way from the sternum into the trachea. One thing, however, was clear, both from the embarrassed respiration and from the change in the character of his voice,—viz. that whatever mischief there might be in the trachea, still that there must be much in the larynx.

The threatenings of suffocation had been returning nearly every night for a week, and often once or twice during the day, when I was summoned, early in the morning of the 25th September, to see him immediately, for he was believed to be dying. When I arrived, I found him in some measure recovered from the most severe attack he had hitherto experienced:

he was sensible, but his breathing was very laborious and difficult. Venesection afforded him some relief.

As I was not at this time provided with a proper cannula to introduce into his trachea, (certainly a great remissness on my part,) I left him for the purpose of having one made, and promised to see him again at three o'clock in the afternoon; and, unless some very decided amendment occurred by that time, (which I could not at all anticipate,) I should be ready to operate upon him. I arrived at his house precisely at the appointed time, and learnt that his family had been sending about for me in all directions, for upwards of an hour; for he had been again attacked so severely, that it appeared as if every breath would be his last; and they even told me, on my arrival, that I was too late, for they actually believed him to be dead. I found him supported in a chair, completely insensible. I positively could feel no pulse; and, as to his respiration, it seemed to be a matter of doubt whether he had not breathed his last. His face was suffused with blood, and his lips perfectly livid. I lost not a moment in dragging him to a window, and laying open his trachea, by an incision which divided half an inch of the lower border of the thyroid gland, and extended downwards for an inch and a half towards the sternum, from which it was distant three-quarters of an inch. A large thyroid vein came in the way of my incision, but I was enabled to avoid wounding it: indeed, I was not at all incommoded by hemorrhage, even from the divided thyroid gland. A longitudinal incision in the front of the trachea instantly gave entrance to the atmospheric air; but I very quickly formed a freer passage, by cutting out with my scalpel a circular piece, half an inch in diameter. His respiration rapidly improved; so that, from being drawn at long intervals, the number of respirations soon amounted to twenty in a minute. After he had continued to breathe freely through the opening for about twenty minutes, I introduced the cannula, which, as I expected, excited a violent convulsive cough. I immediately withdrew it, and a quantity of bloody mucus was expelled. In half an hour from the operation, the patient became quite sensible for a minute or two, but again relapsed into his former state of insensibility, although the breathing went on with the utmost freedom through the aperture. The cannula was again introduced, and suffered to remain for two or three minutes. Less irritation was excited, but enough to produce the expectoration of an additional quantity of mucus.

I now passed a probe down the cavity of the trachea, as far as its division into the bronchi, without exciting any particular pain or irritation. I also passed my finger along the front of the trachea, and felt the innominata crossing it, and pulsating with vehemence, within half an inch of the aperture. In three-quarters of an hour, I again introduced the cannula, and secured it in the trachea, by tapes passing round the neck, and tied behind. The edges of the wound were brought together with adhesive plaster. I now tried the effect of closing the orifice of the cannula with my finger; but, had I persisted long in this experiment, I should soon have brought my patient to his former condition.

At nine in the evening, I found him respiring very easily through the cannula. He had coughed three or four times, but without suffering any great distress.

26th.—Has passed a very good night, having been disturbed only two or three times by coughing. His breathing is free, his countenance improved, and he swallows without difficulty. Cannot speak so as to be heard, without closing the tube, when his voice sounds like a loud whisper. Pulse ninety.

27th.—Tube clogged a little by mucus; respiration consequently less free; speech notwithstanding improved; pulse good; bowels clear.

28th.—Breathes with perfect freedom through the cannula, notwithstanding some slight accumulation of mucus within it, which is easily removed by coughing. By closing the orifice of the cannula with his finger, he can now speak in his natural tone of voice, as distinctly as he has done for three weeks before the operation. The wound above and below the passage for the cannula is healed. There appears no objection to his resuming the use of mercury, and he is therefore directed to take of Pil. hydrarg. gr. x.; Ext. opii gr. j. o. n.; Pil. hydrarg. gr. v. o. m.

30th.—The condition of the larynx is so much improved, and the passage of the air through it so free, that he can now utter a loud and intelligible whisper, even without closing the tube.

October 4th.—Improving daily. The tube has been hitherto removed, and cleared every day.

10th.—Cannula removed but every other day. The mucus is easily detached by a hooked probe, when it becomes at all troublesome and inspissated. The passage is so contracted, that, after the cannula has been withdrawn, it is with difficulty re-introduced: its entrance is, however, much facilitated by passing a bougie through it, the rounded end of which projects a little beyond the edge of the cannula. I had anticipated some difficulty in withdrawing and replacing the cannula, on account of its curved form, so necessary in order that it might remain easily in the trachea, without exciting irritation in that canal, at the same time that it afforded the freest possible ingress and egress to the air; and I should, in consequence, have made use of a double cannula, and have withdrawn the inner one to clean it, while the outer retained its place, had not its inflexibility and its curved form rendered this obviously impossible. However, I had no difficulty in passing it after I employed the bougie.

20th.—Continues to gain ground. Tube removed every third day. Voice more distinct. His mouth being slightly touched by the mercury, he takes but five grains pil. hyd. h. s.; has resumed the decoct. sars. c.

November 1.—Keeps the tube stopped with a cork for half an hour together, while he breathes through the larynx with perfect freedom.

December 1.—The quantity of mucus collected in the tube is now so much lessened, that the tube is removed but once in five or six days. His voice is somewhat raucous. He suffers not the slightest inconvenience from the tube; and is in much better health than he has been for many months past. He has discontinued the mercury, but takes the decoct. sarsæ. c. daily.

1824. January 1st.—He is now so completely recovered, that I should withdraw the tube altogether, did I not apprehend a probability of some inflammation returning in the throat, from the frequent exposure to cold and night air to which his employment subjects him; and, as the tube creates but little trouble, being removed only once a week, I have thought it better to let it remain in the trachea until the winter is past. He takes no medicine of any sort.

June 17th.—He continues perfectly well, and has had no occasion for medicine since the last report. The cannula has been cleaned about once in a fortnight. I withdrew the tube, and inserted a dossil of lint half-way down the wound, to keep its edges apart until the opening in the trachea should be closed.

20th.—The wound barely admits a probe.

21st.—The wound completely healed; and, now that all the air passes freely through the glottis, his voice is as strong and as distinct as it has been for some years past; and his health is perfectly re-established.

The above account was drawn up, and I was on the point of forwarding it for insertion in your Journal, when I saw the report of Mr. Carmichael's case of tracheotomy, performed in the Whitworth Hospital, Dublin, on a patient of Dr. Crampton's with laryngitis, and copied in the Medical and Physical Journal for September; which report I am induced to notice, because, although the operation appears to have been very adroitly performed, and very successful in its issue, I cannot help dissenting from Mr. Carmichael in his condemnation of the tube for tracheotomy, which, he says, "is objectionable on two accounts, and not attended with any advantage: for, first," says he, "a tube excites intolerable irritation, with violent fits of coughing; and, secondly, the apertures in it cannot be sufficiently large to permit the expulsion of the viscid phlegm, which accumulates in most of the cases where tracheotomy is necessary."

Now, as objections to the use of the tube, coming from such high authority, might have an influence on the minds of the junior members of our profession, I feel it incumbent on me to attempt to controvert them; and the rather as I am enabled to cite another case I have seen, in addition to the one I have detailed, in which the tube was employed, without giving rise to those inconveniences which Mr. Carmichael ascribes to its use. The case I allude to occurred about seven years ago, in the person of a silversmith at Portsea, on whom the operation had been performed (I believe) by Dr. Denmark, of Haslar, on account (as I understand) of cynanche tonsillaris. When I saw the patient, he had worn the tube for many weeks, and was in much the same condition with my patient about two months after the operation. He made no complaint whatever of any suffering caused by the tube.

I do not know whether Mr. Carmichael means that his objections should apply to the straight cannula. If he does, I should, to a certain extent, agree with him, as it would be very difficult to introduce a straight tube far enough into the canal to be secure, without risk of its extremity frequently rubbing against the posterior surface of the trachea, in the movements of that organ, and thereby irritating its highly sensible membrane, and perhaps producing violent fits of coughing: though, even in such a case, I see no reason why the aperture could not be sufficiently large to permit the expulsion of mucus, &c. If an opening be made into the trachea large enough to admit the little finger, surely a cannula of the same size may be passed in; the thickness of the metal (silver) not lessening the aperture very materially.

But, with regard to the curved tube, I conceive Mr. Carmichael's objection does not at all apply; for, from its very form, it will, when it has once been introduced, lie quietly along the trachea, having its smooth and polished side in contact with the membrane, and with no projecting edges to create irritation; while its aperture, being in the direction of the axis of the trachea, will afford the freest possible passage both to air and mucus. It is far less liable to displacement than the straight tube, in the ascent and descent of the trachea; and it has an advantage in common with the straight tube,—viz. that, if it fills the aperture made in the trachea, it will prevent the blood (which occasionally continues for a long while to ooze from the thyroid vessels,) from finding its way into the trachea, and thereby creating great distress and severe coughing. Of course, in some instances the patient might be put into such a position as to avoid this inconvenience; but, where that is not expedient, the advantage of the curved cannula will be apparent. However, facts must speak for themselves; and I do not believe I have much more to do, in the present instance, than briefly to advert to the report of J. L.'s case, where, except at the moments of its first and second introduction, the tube did not excite irritation or violent coughing; and where the aperture was sufficiently large to permit, not only the free passage of air, but also the expulsion of mucus; for, although a small portion necessarily adhered to the sides of the tube, and there became inspissated, yet the greater part of what was secreted was constantly passing through it with facility.

I cannot quit the subject without observing, that I found no need of scissars to enlarge the aperture in the trachea, and found no difficulty in removing a circular piece with my scalpel, of a proper size to admit the cannula.

I am glad to observe the mention of several cases of tracheotomy lately, as I am convinced it is an operation that might be very advantageously performed in many cases where it has not been hitherto undertaken.

P.S.—I annex a sketch of the tube I used.

*Clifton; May 12th*, 1825.

## Figures and Tables

**Figure f1:**